# Single qubit neural quantum circuit for solving Exclusive-OR

**DOI:** 10.1016/j.mex.2021.101573

**Published:** 2021-11-06

**Authors:** I.V. Grossu

**Affiliations:** University of Bucharest, Faculty of Physics, Bucharest-Magurele, P.O. Box MG 11, 077125, Romania

**Keywords:** Perceptron, Quantum artificial neural networks, Linearly nonseparable problems, Backpropagation, Quantum computing, Artificial intelligence

## Abstract

The main goal of this work was designing a single qubit neural quantum circuit for performing Exclusive-OR, a concrete example of an operation requiring multiple layers in classical neural networks. The corresponding Qiskit code was tested on both simulators and IBM's “ibmqx2” five-qubit quantum processor. Further analyses along training the proposed neural quantum circuit are currently in progress.

• A single qubit neural quantum circuit for performing XOR was tested on IBM Q Experience

• XOR is a concrete example of an operation requiring multiple layers in classical neural networks

## Graphical abstract

**Figure:**
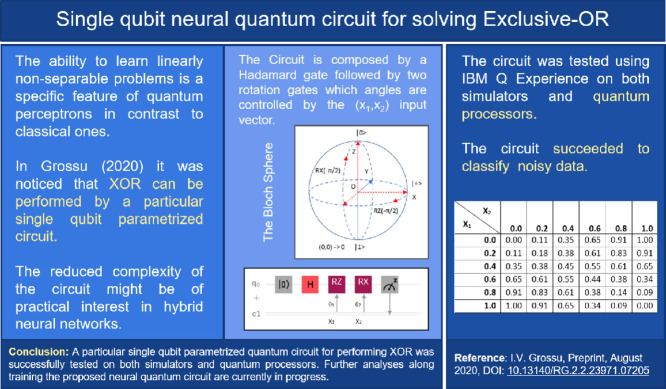

Specifications table

**Table:** 

Subject Area:	Computer Science
More specific subject area:	Artificial intelligence
Method name:	Solving XOR with a single qubit neural quantum circuit
Name and reference of original method:	*Not applicable*
Resource availability:	*Not applicable*

## Introduction

A perceptron [1,2] is a mathematical model inspired by neural cells. It accepts *n* input values “connected” to a single output. Each input channel is characterized by a weight parameter. The output is calculated using the activation function:

(1) $$y=\left\{ \begin{array}{cc} 1, & if\;\sum_{i=1}^{n}x_{i}w_{i}>t,\\ & 0,\;\;else \end{array}\right.$$

where *y* is the output, *x* the input vector, *w* the trainable weight vector, and *t* a threshold trainable parameter.

Although of high interest in both neurosciences and artificial intelligence, the model was proven to be limited to linearly separable functions [2]. Thus, for solving the Exclusive-OR (XOR) function a multi-layer perceptron should be considered instead (the connectionist paradigm).

In the field of Quantum Neural Networks there are various attempts [3], [4], [5], [6]–7] at implementing quantum perceptrons. One important direction is based on the qubit neuron concept (“quron”). Most models are exploiting the nonlinearity of the measurement process for implementing the threshold function. Another direction is based on mimicking the classical Rosenblatt perceptron. As opposed to the classical case, quantum perceptrons are not limited to linearly separable problems.

Hybrid quantum-classical neural networks are described in [8]. The approach is based on implementing hidden layers with parameterized quantum circuits (circuits which rotation angles for each gate are specified by the components of a classical input vector). The measurement statistics of each quantum layer is collected and used as input for the following layer. One could calculate the gradient as the difference between the circuit evaluated at *θ + s* and *θ – s*, where *θ* represents the circuit parameters, and *s* is a macroscopic shift. Thus, the circuit could be trained using gradient descent methods, such as backpropagation. The detailed method, including PyTorch code examples, is also discussed in Ref. [8].

The ability of single quantum neuron to compute linearly non-separable functions is directly connected with the basic property of quantum mechanics that a wave function is invariant under application of global phase [3,6]. For the same reason and, as opposed to classical neurons, AND and OR operations are non-trivial for single quantum neurons [7]. Thus, in Siomau's model [3] is discussed the possibility of computing XOR with a two-qubits perceptron. In Ref. [9], it was noticed that XOR can also be performed by a particular single qubit parametrized circuit.

## Circuit implementation

A single qubit neural circuit is presented in [8]. It contains a Hadamard followed by a rotation *RY(θ)* gate, where *θ* is a trainable parameter. The output in z-basis is given by the expectation:

(2) $$\sigma =\sum_{i}z_{i}p\left(z_{i}\right)$$

where *p(z_i_)* represents the probability of measuring the value *z_i_*.

**Fig. 1:**
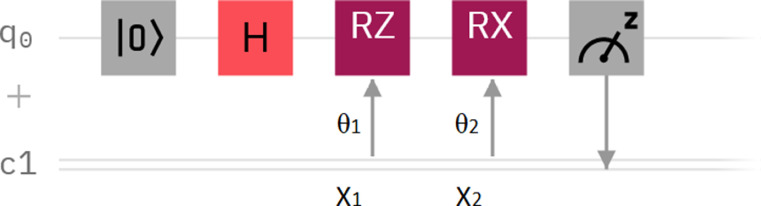
Single qubit parametrized quantum circuit for solving XOR.

Starting from this example, a particular parametrized quantum circuit with two rotation gates was considered (Fig. 1): RZ(θ_1_ * x_1_ + α), and RX(θ_2_ * x_2_ + α), where (*x_1_, x_2_*) is the input vector, (*θ_1_, θ_2_*) are the “dendrites” trainable weights, and *α* is also a trainable parameter. The Hadamard gate is used for initializing the qubit to the |+> diagonal state, while the angles of the two rotation gates are controlled by the (*x_1_, x_2_*) input vector. The output is obtained by collecting the measurement statistics for calculating the expectation *σ* (Eq. (2)). The corresponding Qiskit code is also presented in Appendix A.

**Fig. 2:**
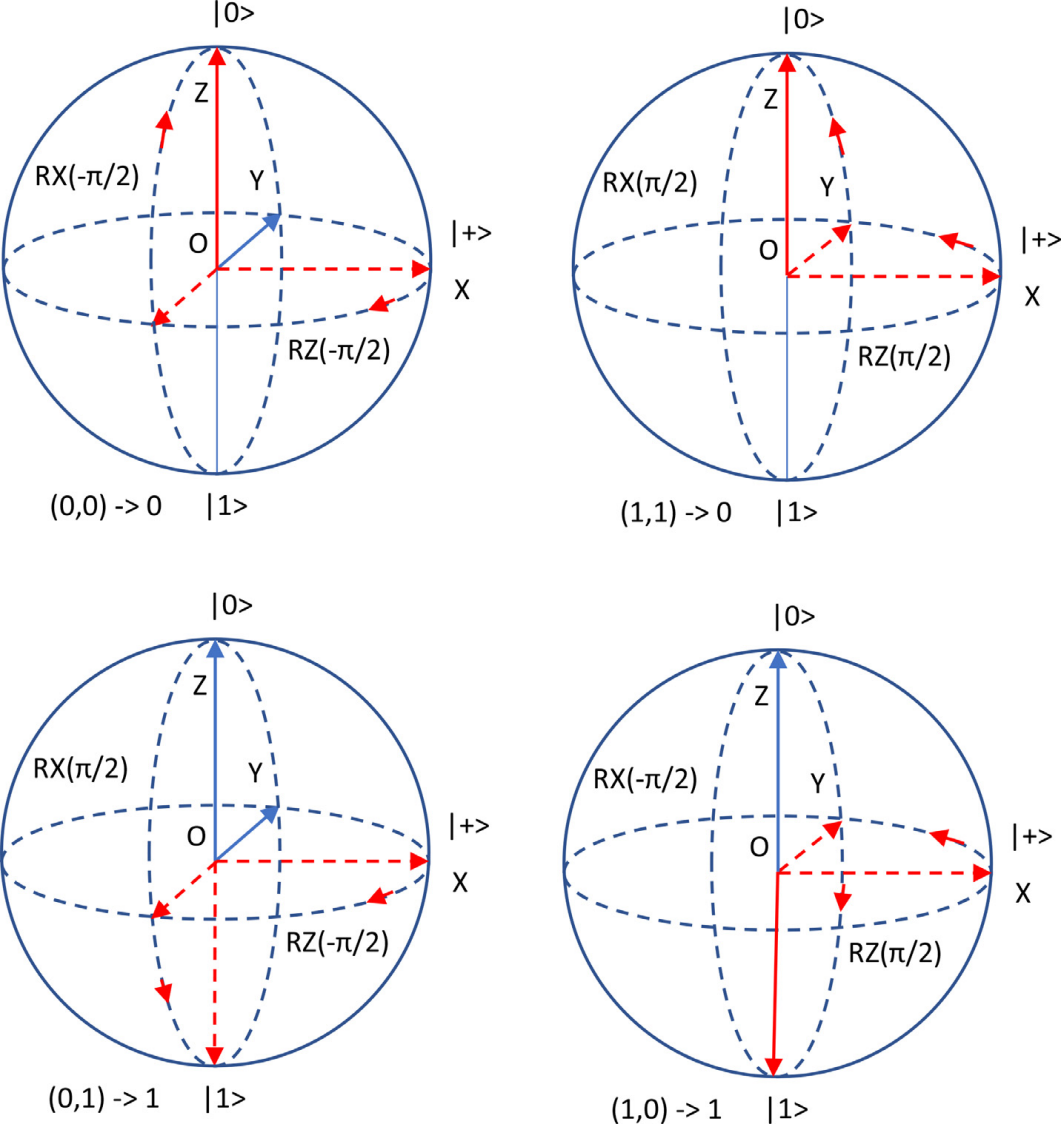
Single qubit XOR solution illustrated on the Bloch Sphere.

The circuit could be trained using gradient descent methods as it was designed following the parametrized quantum circuits principles described in [8]. However, for the XOR problem, one solution could be immediately observed on the Bloch Sphere [10]: *θ_1_= θ_2_=π,* for *α = -π/2*. This case is illustrated, for each input vector, in (Fig. 2).

## Testing the circuit

The circuit was implemented on IBM Quantum Composer [11] which provides a user-friendly graphical development environment, and which allows running the generated OpenQASM code on several quantum computers(an example of code is available in Appendix B). For a better understanding, both the “ibmq_qasm_simulator” simulator and the “ibmqx2” five-qubit quantum processor were chosen. The results are presented in Table 1. The differences between the simulator and quantum computer outputs are due to quantum errors [8]. For evaluating the circuit behavior on input data affected by errors, the attached Qiskit code (see Appendix A) was employed on IBM Quantum Lab. One can notice that, within a reasonable error, the circuit succeeded also to classify noisy data (Table 2).

**Table 1:** The XOR circuit test results.

x_1_	x_2_	32q ibmq_qasm_simulator	5q ibmqx2
0.0	0.0	0.0000	0.0585
0.0	1.0	1.0000	0.8805
1.0	0.0	1.0000	0.8875
1.0	1.0	0.0000	0.0585

**Table 2:** The XOR circuit test results for input data affected by errors.

**X_1_**	**X_2_**	**0.0**	**0.2**	**0.4**	**0.6**	**0.8**	**1.0**
**0.0**		0.00	0.11	0.35	0.65	0.91	1.00
**0.2**		0.11	0.18	0.38	0.61	0.83	0.91
**0.4**		0.35	0.38	0.45	0.55	0.61	0.65
**0.6**		0.65	0.61	0.55	0.44	0.38	0.34
**0.8**		0.91	0.83	0.61	0.38	0.14	0.09
**1.0**		1.00	0.91	0.65	0.34	0.09	0.00

## Conclusion

A particular single qubit parametrized quantum circuit for performing XOR was successfully tested on both simulators and the “ibmqx2” five-qubit quantum processor. Further analyses along training the proposed neural quantum circuit are currently in progress.

## Direct submission or co-submission

Co-submissions are papers that have been submitted alongside an original research paper accepted for publication by another Elsevier journal

## Declaration of Competing Interest

The authors declare that they have no known competing financial interests or personal relationships that could have appeared to influence the work reported in this paper. The authors declare the following financial interests/personal relationships which may be considered as potential competing interests: The author declares no conflict of interest.

## Appendix

A. The Qiskit code used for testing the XOR circuit on IBM Quantum Lab:

*# importing standard Qiskit libraries and configuring account*from qiskit import QuantumRegister, ClassicalRegister, QuantumCircuit, execute, Aer, IBMQ from qiskit.compiler import transpile, assemble from qiskit.tools.jupyter import * from qiskit.visualization import * from numpy import pi qreg_q = QuantumRegister(1, 'q') creg_c = ClassicalRegister(1, 'c') circuit = QuantumCircuit(qreg_q, creg_c)

*# the input vector is manually changed on each run* x1 = 0.0 x2 = 1.0

*# hardcoded trainable parameters* theta1 = pi theta2 = pi alpha = -pi/2

*# parametrized quantum circuit* circuit.reset(qreg_q[0]) circuit.h(qreg_q[0]) circuit.rz(x1 * theta1 + alpha, qreg_q[0]) circuit.rx(x2 * theta2 + alpha, qreg_q[0]) circuit.measure(qreg_q[0], creg_c[0])

*# the simulator to be used* sim = Aer.get_backend('aer_simulator')

*# turns the circuit into an object the backend can run* qobj = assemble(circuit, shots=1024)

*# run the experiment and get the result from that experiment* result = sim.run(qobj).result()

*# get a dictionary containing the output* counts = result.get_counts()

*# display output on a histogram* plot_histogram(counts)

B. An example of OpenQASM 2.0 code generated with IBM Quantum Composer:

OPENQASM 2.0; include "qelib1.inc"; qreg q[1]; creg c[1]; reset q[0]; h q[0]; rz(-0.5*pi) q[0]; rx(-0.5*pi) q[0]; measure q[0] -> c[0];

## Appendix Supplementary materials

Image, application 1

Image, application 1
